# Microwave Hyperthermia of Brain Tumors: A 2D Assessment Parametric Numerical Study

**DOI:** 10.3390/s22166115

**Published:** 2022-08-16

**Authors:** Jan Redr, Tomas Pokorny, Tomas Drizdal, Ondrej Fiser, Matous Brunat, Jan Vrba, David Vrba

**Affiliations:** Faculty of Biomedical Engineering, Czech Technical University in Prague, 160 00 Prague, Czech Republic

**Keywords:** hyperthermia, microwave hyperthermia, brain cancer, SAR

## Abstract

Due to the clinically proven benefit of hyperthermia treatments if added to standard cancer therapies for various tumor sites and the recent development of non-invasive temperature measurements using magnetic resonance systems, the hyperthermia community is convinced that it is a time when even patients with brain tumors could benefit from regional microwave hyperthermia, even if they are the subject of a treatment to a vital organ. The purpose of this study was to numerically analyze the ability to achieve a therapeutically relevant constructive superposition of electromagnetic (EM) waves in the treatment of hyperthermia targets within the brain. We evaluated the effect of the target size and position, operating frequency, and the number of antenna elements forming the phased array applicator on the treatment quality. In total, 10 anatomically realistic 2D human head models were considered, in which 10 circular hyperthermia targets with diameters of 20, 25, and 30 mm were examined. Additionally, applicators with 8, 12, 16, and 24 antenna elements and operating frequencies of 434, 650, 915, and 1150 MHz, respectively, were analyzed. For all scenarios considered (4800 combinations), the EM field distributions of individual antenna elements were calculated and treatment planning was performed. Their quality was evaluated using parameters applied in clinical practice, i.e., target coverage (TC) and the target to hot-spot quotient (THQ). The 12-antenna phased array system operating at 434 MHz was the best candidate among all tested systems for HT treatments of glioblastoma tumors. The 12 antenna elements met all the requirements to cover the entire target area; an additional increase in the number of antenna elements did not have a significant effect on the treatment quality.

## 1. Introduction

Hyperthermia (HT) treatments, characterized as an artificially induced temperature rise of 40–44 °C typically for a duration of 60–90 min, have been proven to increase the survival rates of patients with a wide range of tumors when combined with radiotherapy (RT) [1] and/or chemotherapy (CHT) [2]. Combining HT treatments with standard modalities for brain tumor treatments can be of great benefit as RT can lead to severe toxicity, especially in children [3]. A double increase in the 2-year survival rate (from 15% to 31%) was shown for a combination of an HT treatment with RT versus RT alone for 112 patients with recurrent head tumors, including glioblastomas, squamous cell carcinomas, or melanomas [4]. An increase in the 2-year survival rate from 18% to 47% in patients with higher-grade gliomas was demonstrated in [5]. Man et al. [6] demonstrated an increased radiosensitivity of glioma stem cells, which contribute to tumor progression by resisting radiation therapy, when these cells were treated with a combination of an HT treatment and RT versus RT alone. Despite these promising results, HT treatments for brain cancer are not fully established due to technical difficulties. 

The temperature increase in a tumor in microwave HT treatments is generally induced by the constructive interference of EM waves irradiating from applicators with a phased array consisting of antenna elements surrounding the treated area. Applicators operating from 434 to 915 MHz are the most common for HT head and neck (the area including the neck and lower half of the head outside the brain) cancer treatments due to their ability to penetrate human tissues and generate a controlled heat focus within the tumor region [3]. There are several head and neck HT applicators, consisting of different numbers of antenna elements and operating at different frequencies. HYPERcollar and renewed HYPERcollar3D applicators operate at a 434 MHz frequency and use 12 antenna elements in a 2-ring configuration or 20 antenna elements in 3-ring configuration, respectively [7,8]. Rodriguez et al. developed an annular phased array operating at a 915 MHz frequency with 72 antenna elements in a 3-ring configuration [9]. 

There are several key design parameters in the optimization of HT phased array applicators; the most prominent are the operating frequency and the number of antenna elements. The relationship between these parameters is non-linear and their impact on the HT treatment performance is complex. Guérin et al. [10] demonstrated that the most suitable frequency for a head and neck HT system was higher than 900 MHz with as high a number of antenna elements as possible. Takook et al. [11] described a similar trend; increasing the number of antennae operating at higher frequencies led to better power absorption in the tumor than using fewer antenna elements operating at lower frequencies. On the contrary, Paulides et al. [12] found that the optimal frequency was 434 MHz with 8 antenna elements placed in a 1-ring configuration. The studies above show that there is no clear consensus regarding head and neck HT system synthesis and optimization in terms of operating frequency and the number of antenna elements.

The purpose of this study was to evaluate the effect of the operating frequency and the number of antenna elements of a regional HT phased array system on the ability to create a clinically relevant distribution of the specific absorption rate (SAR) in and around the treatment target. For this purpose, we: (a) created a total of 4800 2D numerical models (scenarios) differing in anatomically and geometrically realistic patient-specific head models as well as the diameter and position of the tumor model, the number of antenna array elements, and the operating frequency (Section 2.1); (b) calculated the electromagnetic (EM) field distribution in the head models from all individual antenna elements for four pre-selected operating frequencies (Section 2.2); and (c) planned hyperthermic treatments according to current clinical practice and evaluated the treatment planning quality (Section 2.3).

## 2. Methods

### 2.1. The 2D Head Phantom Models

**Figure 1:**
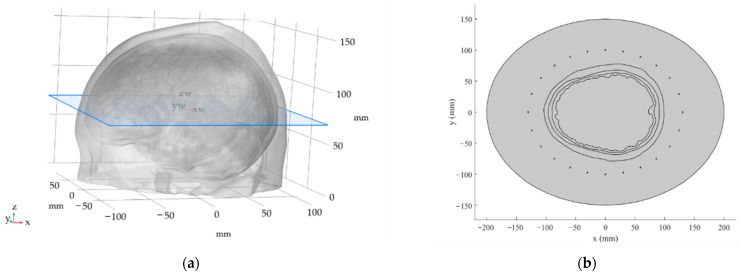
The 3D head model and derivation of 2D geometry using planar cross-section (blue plane) located at the widest point of the 3D head model (**a**) and the 24-antenna element HT system. Antenna ele-ments are represented by a circular cross-section placed on the circumference of the ellipse (major and minor half-axis equal to 130 mm and 100 mm, respectively). The distribution of domains from the center of the head is as follows: brain tissue; cerebral spinal fluid (CSF); skull; and scalp domain. The deionized water domain is represented by an elliptical surface (major and minor half-axes are equal to 200 mm and 150 mm, respectively) surrounding the head model (**b**).

Initially, 10 realistic 2D head geometries were created. These geometries were derived from 3D head models obtained from [13]. The 3D models were imported into COMSOL Multiphysics [14] using Materialize 3-matic software [15]. The 2D models were then created as a planar cross-section that was set in the widest part of the respective head model (Figure 1a). The geometries of all 10 head models are depicted in Appendix A.

We proposed two numerical models of the HT phased array applicator; the first model consisted of 24 antenna elements and the second model consisted of 16 antenna elements. The antenna elements were defined as a circular cross-section with a radius of 1 mm placed in an elliptically-shaped (major and minor half-axis equal to 130 mm and 100 mm, respectively) phased array at an average of 3 cm from the surface of the head model. Henke et al. [16] showed that an equidistant distribution of the antenna elements performed the best with a symmetrical setup, which is approximately the case of the brain. The 24-antenna element HT applicator is depicted in Figure 1b.

#### Hyperthermia Targets: Size and Position

A study [16] concluded in Finland on 331 adults provided the incidence of the most recurring tumors according to their most frequent positions in the brain. In addition to a location analysis, the study concluded that patients suffering from glioma brain tumors developed a multiform glioblastoma with the highest probability (47%). The second most common glioma tumor was an astrocytoma, with an incidence of 23%.

Tumors are often diagnosed in the following brain areas:

1. Frontal lobe, with an incidence of 40%;
2. Temporal lobe, with an incidence of 29%;
3. Parietal and occipital lobe, with an incidence of 17%;
4. Deep-seated tumors, with an incidence of 14%.

**Figure 2:**
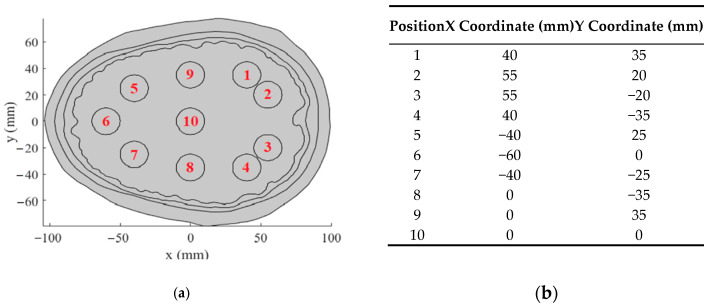
Hyperthermia target position layout in a 2D model (**a**) with the corresponding coordinates in a table (**b**).

We reserved seven out of ten hyperthermia target positions (positions 1–7) selected in our analyses for the frontal and occipital lobe. Two positions (8 and 9) were chosen for the parietal lobe and one position (10) was chosen for the central position. The hyperthermia target position layout can be seen in Figure 2a; the coordinates relative to the center of the brain are listed in the table in Figure 2b.

In this study, we used spherical hyperthermia targets of three different diameters: 20, 25, and 30 mm. The chosen diameters were on the smaller side of the tumor diameters presented in [17]. Smaller hyperthermia targets present a more challenging situation in HT treatment planning as it is harder to focus microwave energy in smaller volumes [10]. Table 1 summarizes the diameters of the hyperthermia targets.

For the most frequent locations of head tumors, analyses of the frequency as well as the number of antenna elements were performed. The hyperthermia targets were placed in the three most recurrent lobes—the frontal, temporal, and occipital—at approximately the same depth whereas the hyperthermia targets in the central part of the brain were located from 8 to 10 cm beneath the skin.

### 2.2. Numerical Simulations 

In this study, a well-proven numerical full-wave EM field simulator, COMSOL Multiphysics [14], based on the finite element method (FEM) was used to calculate the distribution of the electromagnetic field in the head area of each individual antenna element. Due to the simplification to 2D, the considered electromagnetic waves were out-of-plane TEz with field components Ez, Hx, and Hy. The boundary condition applied to the outer edge of the computing area was the scattering boundary condition (BC) plane wave. This BC suppressed the reflection of the EM waves when they hit the inner edge of the computational area. As a result, the computational area could be reduced to the minimum size necessary to accurately calculate the EM field distribution in the head area. The boundary condition port was used on the surface of the circles representing the antenna elements; the electric field of the port was set only for the z-component.

**Figure 3:**
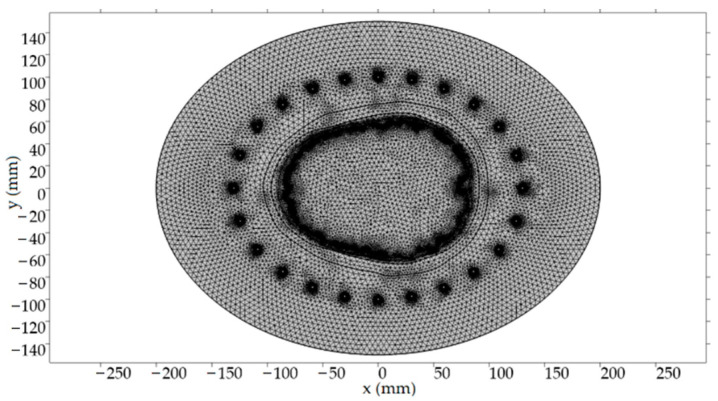
Discretization mesh of one of the considered scenarios (for illustrative purposes only).

A typical triangular mesh for the FEM was used to discretize the computational area. The maximum value of the side length of the triangular mesh element was set to 1/8 of the transversal EM wave wavelength in the given environment and for the highest considered frequency (the manufacturer’s recommendation is 1/5). Table 2 shows the general mesh settings used for all our models. The numbers of the domain and boundary elements for the model of Patient No. 1 were 52,858 and 2546, respectively. The discretization mesh for one of the considered scenarios is shown in Figure 3.

The dielectric parameters of the domains representing the biological tissues and the water bolus for the frequencies considered are given in Table 3. Their values were based on the frequency-dependent 4-pole Cole–Cole model [18]; its parameters for individual biological tissues and water are available from an online database [19]. The domain forming the inner part of the antenna elements was excluded from the computational area because it was surrounded by the port boundary condition and thus did not influence the simulation results.

In addition to the 434 and 915 MHz frequencies commonly used in hyperthermia treatments, two others with values approximately 200 MHz above these frequencies were included in this study. We performed numerical simulations for 10 head geometries with a 24-antenna element system and 16-antenna element system, respectively, at frequencies of 434, 650, 915, and 1150 MHz. The time required for a single 2D simulation was approximately 3 min on a workstation with the following relevant technical specifications: CPU, 2× Intel^®^ Xeon^®^ Silver 4208 (Intel, Santa Clara, CA, USA); GPU, NVIDIA GeForce 2080 (NVIDIA, Santa Clara, CA, USA); and RAM, 192 GB. The electric fields obtained from the simulations were then loaded into MATLAB software [20]. The electric fields for the 12- and 8-antenna element systems were obtained from the 24-antenna element system by taking the electric fields from every second or third antenna element, respectively. From these electric fields, the SAR distributions and optimizations were obtained for all proposed HT systems.

### 2.3. SAR Optimization and Evaluation

The main task during an HT treatment is a controlled temperature increase in the tumor areas, minimally to 40 °C and not exceeding 44 °C in the healthy tissue. Local areas with undesirably high SAR levels resulting in higher temperatures, or so-called hot-spots, may occur in local areas with different dielectric parameters of individual tissues and complex geometries of the treated area.

Ideally, HT treatment planning is based on the calculated temperature distribution in the treated area. However, due to uncertainties in the temperature parameters of biological tissues, temperature calculations are not currently implemented as a standard in clinical practice. 

Bellizzi et al. [21] showed that SAR-derived quantities such as the target to hot-spot quotient (THQ) and the TC_x_ quotient—TC_25_ and TC_50_—can be used as substitutes for the assumed temperature in the head and neck region. For this reason, we used SAR optimization in our study. The target to hot-spot quotient (THQ) of the SAR [8,22,23] is defined as:

(1) $$\mathrm{THQ}\;=\;\frac{{<\mathrm{SAR}}_{\mathrm{target}}>}{{<\mathrm{SAR}}_{1\%\mathrm{HS}}>}$$

where $\langle {\mathrm{SAR}}_{1\%\mathrm{HS}}\rangle$ is the average SAR in 1% of the volume with the highest SAR values outside the target (i.e., the hot-spots) and $\langle {\mathrm{SAR}}_{\mathrm{target}}\rangle$ is the average SAR in the target volume. A THQ above 1 results in a higher SAR in the target than the SAR in the hot-spots. TC_x_ is a percentage of the hyperthermia target volume covered by at least x% of the iso-SAR [8]; for example, TC_50_ is defined as:

(2) $${\mathrm{TC}}_{50}\;=\;\frac{V_{\mathrm{target}}\;(\mathrm{SAR}>0.5\cdot \max\left\{\mathrm{SAR}\right\}\;)}{V_{\mathrm{target}}}\;[\%]$$

where $V_{\mathrm{target}}$ is the target volume in which the SAR is higher than 50% of the maximum SAR in the brain.

We used TC_25_, TC_50_, and TC_75_ as the TC quotients to be evaluated. The TC quotients, together with the THQ, were evaluated for all patient models as a mean value among all the hyperthermia target positions, all frequencies, and all phased array configurations with different numbers of antenna elements. According to [12], TC_25_ can be used to decide whether an HT treatment is possible (in the head and neck region). In addition, TC_25_ was shown to predict the clinical response in a superficial HT treatment for recurrent breast carcinomas [24]. In [25], TC_50_ and TC_75_ were studied to better validate the heating capabilities of the HT system.

For THQ optimization, we applied a PSO (particle swarm optimization) function available in MATLAB [20] with the THQ as a criteria function to be maximized. The PSO returned optimized values for the amplitude and phase settings of the antenna element input signals. A total of 200 iterations were used for the PSO algorithm and a single optimization took around 50 s. In summary, we attempted to achieve:

- The maximization of the THQ, or the reduction of hot-spots;
- The maximization of the target coverage, or an improvement in TC_25_, TC_50_, and TC_75_.

## 3. Results

### 3.1. Operating Frequency

**Figure 4:**
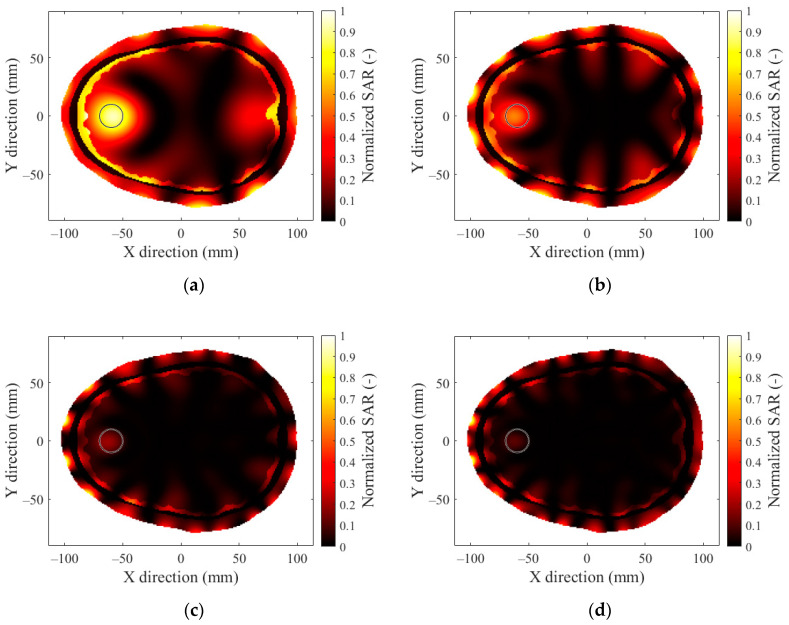
SAR distribution in the 2D head model obtained by a 12-antenna HT system at a frequency of (**a**) 434 MHz, (**b**) 650 MHz, (**c**) 915 MHz, and (**d**) 1150 MHz for hyperthermia target diameter of 20 mm.

The SAR distributions for patient no. 1 with the hyperthermia target located in the frontal lobe 4 cm from the outer layer of the head model and a 12-antenna HT system are presented in Figure 4a for operating frequencies of 434 MHz. Figure 4b presents the results for 650 MHz, Figure 4c presents the results for 915 MHz, and Figure 4d presents the results for 1150 MHz. The target area is marked in Figure 4 with a thin black circle. A set of results comparing the frequencies of the remaining applicators can be found in Appendix B.

**Figure 5:**
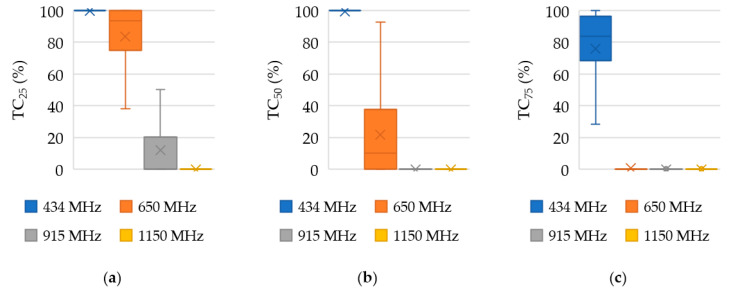
Box plot of (**a**) TC_25_, (**b**) TC_50_, and (**c**) TC_75_ comparing 4 frequencies used for a 12-antenna HT system. The mean values and medians are represented by a cross and a horizontal line, respectively.

A detailed statistical analysis of the TC parameters for the 12-antenna system is presented in Figure 5 as a box plot covering TC_25_ (Figure 5a), TC_50_ (Figure 5b), and TC_75_ (Figure 5c). The lower and upper boundary of the rectangle represents the first (Q_1_) and third (Q_3_) quartiles; the maximum and minimum values are represented with a vertical line facing upward and downward, respectively; and the median and mean values are represented with a horizontal line and a cross, respectively. The values were averaged for 300 samples comprising data from 10 patients, 10 target locations, and 3 sizes of target. 

### 3.2. Number of Antenna Elements

For a better comparison, we present the average values of the TC quotients for the 24-, 16-, 12-, and 8-antenna HT systems in Table 4. The obtained analysis shows that the highest mean TC parameters (in bold) were obtained using the 24- and 16-antenna HT applicator systems operating at a frequency of 434 MHz. However, for this frequency, the 12-antenna applicator provided comparable results with the 16- and 24-antenna applicators.

**Figure 6:**
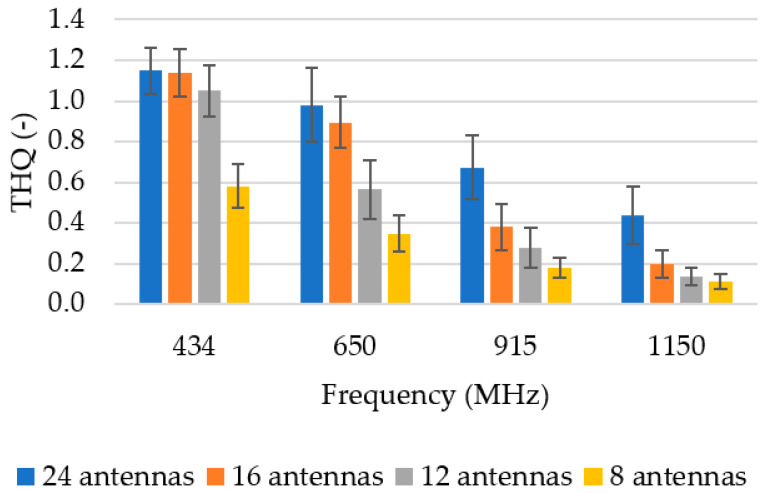
Mean THQ for different frequencies and numbers of antenna elements. Dark lines represent positive and negative standard deviation of the mean.

Figure 6 shows the mean THQ values as a function of the operating frequency and the number of antenna elements. The THQ parameter decreased with an increasing operation frequency and with a decreasing number of antenna elements. However, the goal was to discover the lowest possible number of antenna elements that would sufficiently expose the entire hyperthermia target.

**Figure 7:**
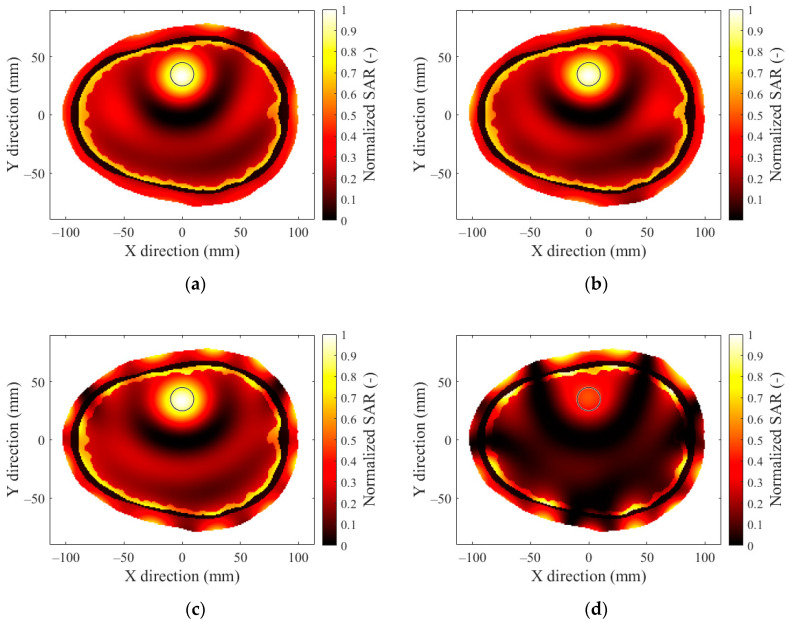
SAR distribution in the 2D head model obtained by (**a**) 24-antenna, (**b**) 16-antenna, (**c**) 12-antenna, and (**d**) 8-antenna HT systems at a frequency of 434 MHz with a hyperthermia target diameter of 20 mm.

In Figure 7, we present the optimized SAR distributions for HT systems with a different number of antenna elements operating at a frequency of 434 MHz, which showed the best TC_25_ in previous operating frequency analyses.

### 3.3. Hyperthermia Target Size

**Figure 8:**
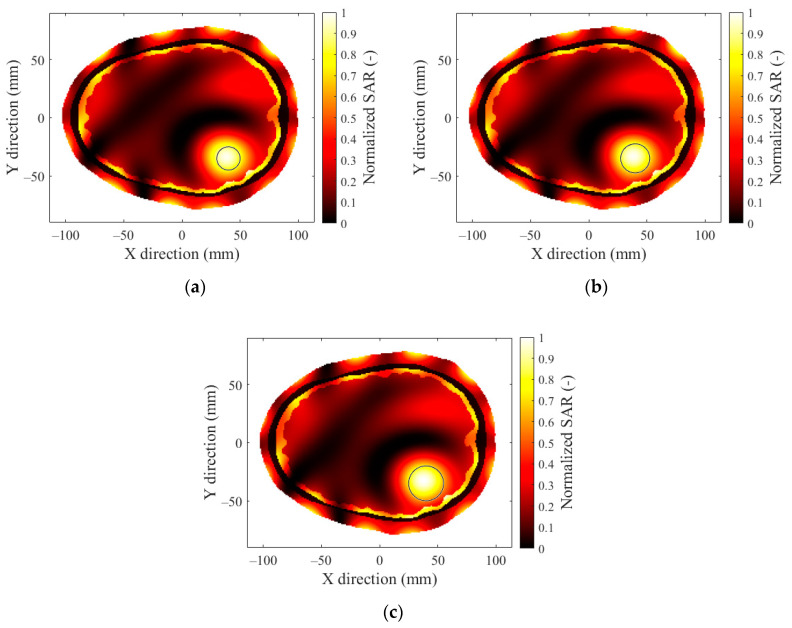
SAR distribution of a 12-antenna HT system at a frequency of 434 MHz for 3 different diameters of hyperthermia targets: (**a**) 20 mm; (**b**) 25 mm; (**c**) 30 mm.

**Figure 9:**
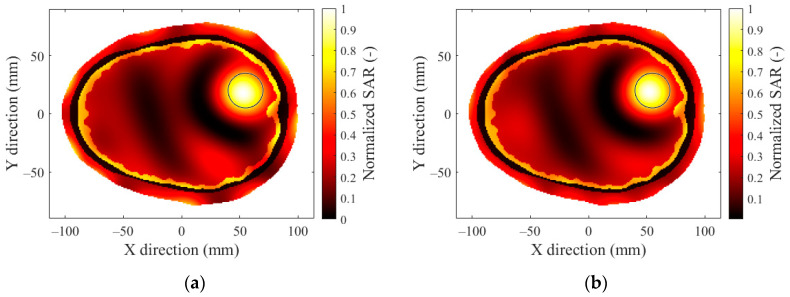

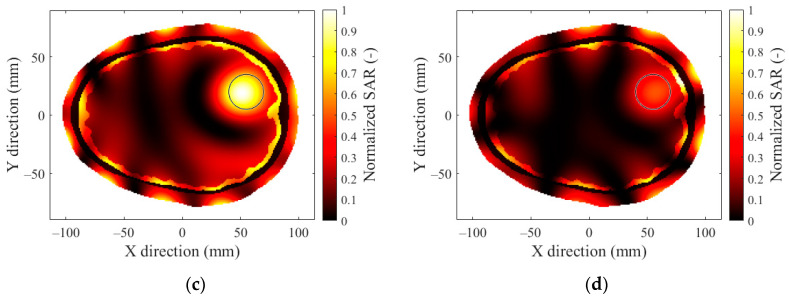
SAR distributions obtained by the (**a**) 24-antenna, (**b**) 16-antenna, (**c**) 12-antenna, and (**d**) 8-antenna HT systems at a frequency of 434 MHz with a hyperthermia target diameter of 30 mm.

**Figure 10:**
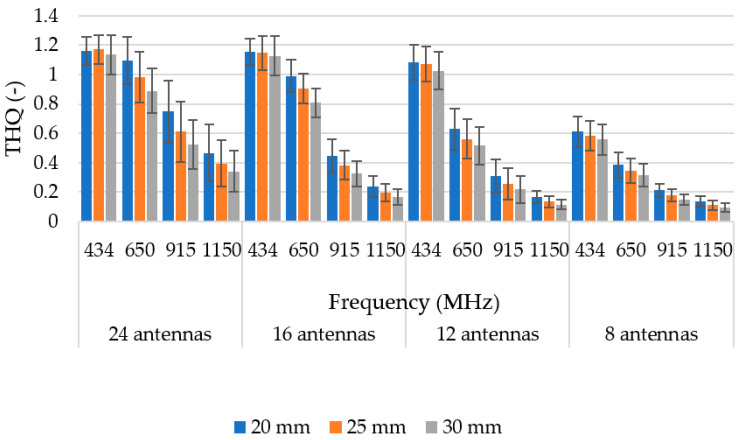
Mean THQ values for 3 different diameters of hyperthermia target. Dark lines represent positive and negative standard deviation of the mean.

**Figure 11:**
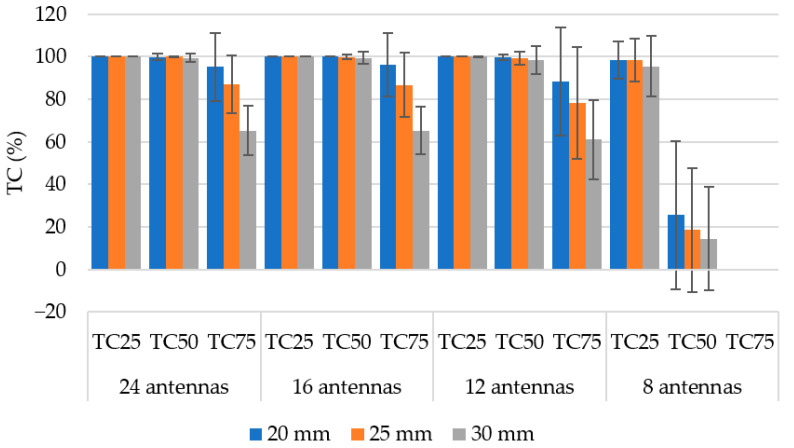
Mean TC values for 3 different diameters of hyperthermia target at a frequency 434 MHz. Dark lines represent positive and negative standard deviation of the mean.

In Figure 8, we present the optimized SAR distributions for a 12-antenna system operating at a frequency of 434 MHz for 3 hyperthermia targets with different diameters. In Figure 9, we present the optimized SAR distributions for systems with different numbers of antenna elements operating at a frequency of 434 MHz with a hyperthermia target diameter of 30 mm. Each hyperthermia target is highlighted by a black circle. In Figure 10 and Figure 11, we present the mean THQ for all frequencies and number of antennae as well as the TC quotients for the 434 MHz frequency, respectively.

### 3.4. Effect of Hyperthermia Target Location

**Figure 12:**
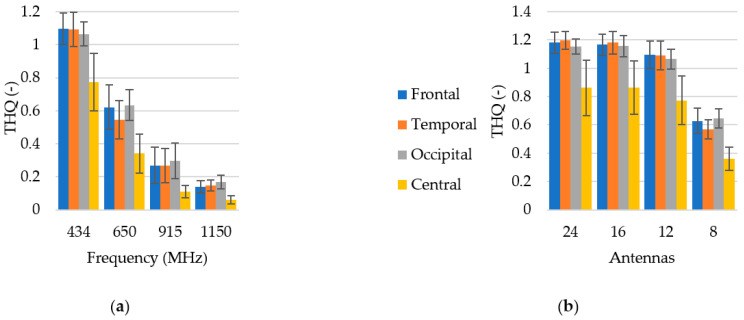
Mean value of THQ for the hyperthermia targets located in the frontal, temporal, occipital, and central part of the brain differing in the frequency of 12-antenna system (**a**) and differing in the number of antenna elements (**b**). Dark lines represent positive and negative standard deviations of the mean values.

In Figure 12, comparisons of the THQ for various hyperthermia target locations and for 4 different frequencies of the 12-antenna HT system (Figure 12a) and for all HT systems operating at a frequency of 434 MHz (Figure 12b) can be seen.

**Figure 13:**
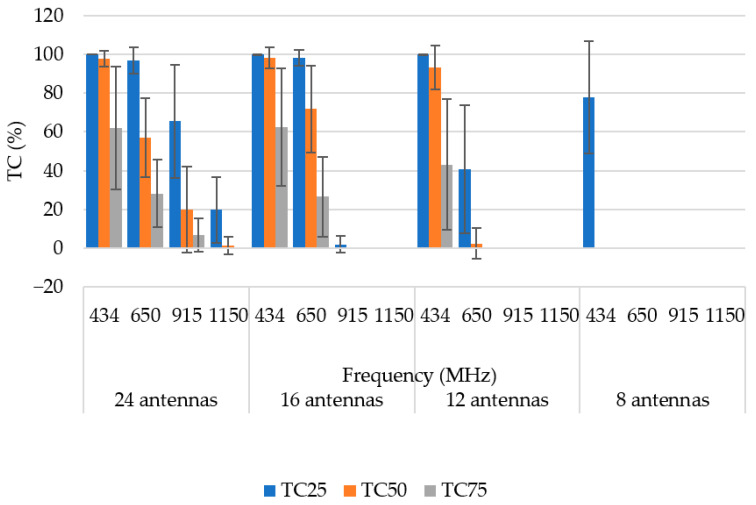
Mean values of TC quotients of the central position of a hyperthermia target for all HT systems and 4 different frequencies. Dark lines represent positive and negative standard deviations of the mean value.

Although the target area located in the central part had the highest hot-spot ratio for every considered frequency, the specific lobes of the brain appeared to have no significant difference in the resulting quality of the HT treatment. From the previous analyses, it was obvious that the lowest frequency offered the best results for every analyzed parameter. In the case of a central position of the hyperthermia target, we provide a TC quotient analysis for every HT system at a frequency of 434 MHz in Figure 13.

**Figure 14:**
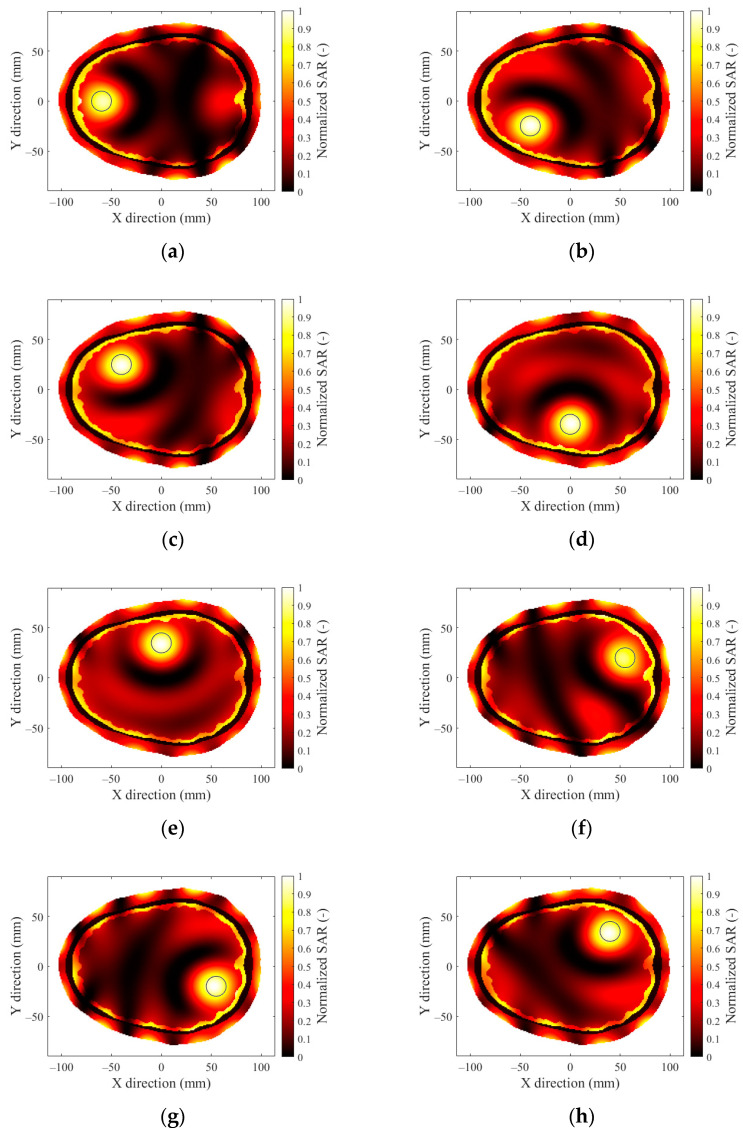

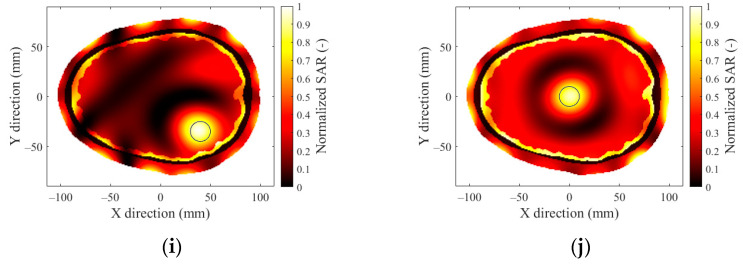
SAR distributions for a 12-antenna HT system operating at a frequency of 434 MHz for all considered positions (**a**–**j**) with a hyperthermia target diameter of 20 mm.

In Figure 14, we present the SAR distribution of the 12-antenna HT system operating at a frequency of 434 MHz; every hyperthermia target position is included.

## 4. Discussion

The 12-antenna phased array system operating at 434 MHz was shown to be the best candidate of all tested systems for the HT treatment of glioblastoma tumors. The simulation results showed that the TC and THQ parameters reached the highest values with a frequency of 434 MHz. These findings were in agreement with the work of Paulides et al. [12] who studied appropriate phased array operation frequencies and number of antennae for neck tumors. However, our results were in conflict with the work of Guérin et al. [10] and Takook et al. [11], who suggested that increasing the number of antenna elements operating at a higher frequency resulted in a better HT treatment of head and neck and brain tumors, respectively. In a clinical environment, a system with low complexity (antenna elements) is preferred [19]. The obtained analysis (Table 4) shows that the highest TC values were reached for the 16- and 24-antenna HT systems operating at 434 MHz. On the other hand (Figure 7), the arrangement with 8 antenna elements showed insufficient SAR values in the target whereas the 12-antenna system provided an almost identical TC_50_ to the 16- and 24-antenna systems. This indicated that increasing the number of antenna elements from 12 to 24 did not affect the quality of the SAR focus. For the 24-, 16-, and 12-antenna systems operating at a frequency of 434 MHz, the THQ and TC parameters were comparable.

The SAR focus quality was affected by the diameter of the hyperthermia targets. To obtain a target conformal SAR focus in smaller hyperthermia targets (below 25 mm) at 434 MHz was difficult due to the wavelength size in comparison with the target dimensions (Figure 8). The SAR focus within the hyperthermia target diameter of 30 mm (Figure 8c) was the best of all studied target diameters (20, 25, and 30 mm). However, even for the 20 and 25 mm targets, the 12-antenna system operating at 434 MHz provided clinically sufficient SAR coverage (TC_25_ > 75%).

The SAR TC depended on the depth of the tumor position rather than its specific area in the head (Figure 12). The 12- and 24-antenna systems operating at 434 MHz ensured the request for the target conformal SAR coverage of the hyperthermia targets located less than 4 to 5 cm below the surface of the scalp. For the hyperthermia targets occurring at a depth of up to 7 cm below the surface, not even a 24-antenna system operating at 434MHz w able to achieve sufficient SAR coverage. For the central hyperthermia target positions (e.g., Position 10 in Figure 2) and all presented HT systems, hot-spots started to occur close to the patient surface, leading to a decrease in the THQ objective function. 

This study has several limitations. First, the use of 2D models: 3D models and simulations are more realistic. We used 2D models due to their lower computational requirements and thus time consumption. We explored thousands of scenarios that would be much more complex for 3D models. On the other hand, 3D models allow for the inclusion of other parameters that have no equivalent in 2D models; for example, the specific geometry of the antenna elements and the water bolus, the orientation of the antenna elements in space, and the corresponding polarization of the EM waves. These would introduce additional variables into this study and could ultimately reduce the generality of the results. Additionally, only numerical simulations without phantom measurements were used. However, from the experience of the authors, the agreement between the calculations and measurements was high in similar cases. The values of the evaluation parameters based on this study corresponded with the values that could be encountered in clinical practice. Lastly, deionized water was used as the matching medium and no other material was taken into account. On the other hand, none of the current HT systems use different materials. This is due to the low loss of EM waves passing through the deionized water, which is important for the low loss of microwave energy when EM waves pass through the matching medium.

## 5. Conclusions

In this study, we investigated the ability of a single-ring HT applicator to deposit EM energy into a target located within the brain by assessing the effect of the HT system operating frequency, the number of antennae, and target position and sizes. We found that a 12-antenna 434 MHz system produced almost identical SAR TCs to the 24- and 16-antenna systems. Due to its lower complexity, we consider this to be the best configuration for HT phased array systems. We plan to expand the investigation with 3D simulations, multiple-ring antenna element configurations, and the testing of our numerical simulations in real-world measurements.

## Figures and Tables

**Table 1:** Hyperthermia target diameters.

Hyperthermia Target Size	Diameter (mm)
Small	20
Medium	25
Large	30

**Table 2:** Maximum and minimum element sizes for every domain used in the computational model.

Tissue	Maximum Element Size (mm)	Minimum Element Size (mm)
Scalp	5.5	0.0225
Skull	9.4	0.09
CSF	4	0.09
Brain tissue	4.9	0.09
Deionized water	5.2	0.09

**Table 3:** Dielectric parameters and densities of domains of computational area. Data obtained from [19].

Tissue	Density (kg/m^3^)	Frequency (MHz)	Dielectric Parameters	
			Electric Conductivity (S/m)	Permittivity (-)
Scalp	1109	434	0.53	38.17
		650	0.58	36.78
		915	0.64	35.88
		1150	0.70	35.37
Skull	1908	434	0.09	13.07
		650	0.12	12.72
		915	0.15	12.44
		1150	0.18	12.23
CSF	1007	434	2.26	70.63
		650	2.32	69.33
		915	2.42	68.60
		1150	2.53	68.18
Brain tissue	1046	434	1.62	48.46
		650	1.70	46.28
		915	1.79	44.86
		1150	1.88	44.06
Deionized water	1000	434	0.04	84.42
		650	0.10	84.37
		915	0.19	84.28
		1150	0.30	84.17

**Table 4:** Mean values of TC_25_, TC_50_, and TC_75_ for a 24-, 16-, 12-, and 8-antenna HT system. Mean TC values in bold represents the highest TC value for a specific frequency.

	Frequency (MHz)	Target Coverage (%)			
		24-Antenna System	16-Antenna System	12-Antenna System	8-Antenna System
TC_25_	434	**100** ± 0	**100** ± 0.02	**100** ± 0.03	97.4 ± 11.35
	650	98.6 ± 4.86	**99.32** ± 2.16	83.8 ± 23	34.28 ± 26.66
	915	**78.56** ± 27.64	41.48 ±27.23	11.83 ± 19.22	0 ± 0
	1150	**51.76** ± 28.95	1.69 ± 5.5	0 ± 0	0 ± 0
TC_50_	434	99.76 ± 1.42	**99.79** ± 1.79	99.22 ± 4.28	19.55 ± 30.08
	650	84.61 ± 16.49	**86.45** ± 14.93	21.59 ± 26.71	0 ± 0
	915	**45.61** ± 24.64	1.76 ± 6.36	0 ± 0	0 ± 0
	1150	**10.99 ± 16.30**	0 ± 0	0 ± 0	0 ± 0
TC_75_	434	82.54 ± 18.71	**82.71** ± 18.88	75.87 ± 26.29	0 ± 0
	650	**45.53** ± 19.66	42.74 ± 21.32	0.45 ± 2.63	0 ± 0
	915	**12.89** ± 13.22	0 ± 0	0 ± 0	0 ± 0
	1150	**1.18** ± 4.61	0 ± 0	0 ± 0	0 ± 0

**Figure A1:**
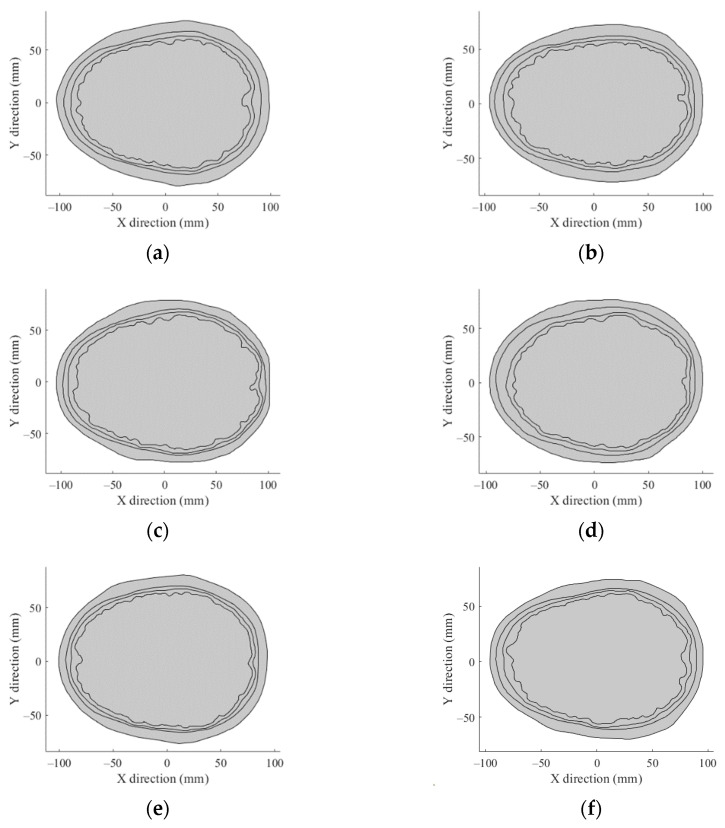

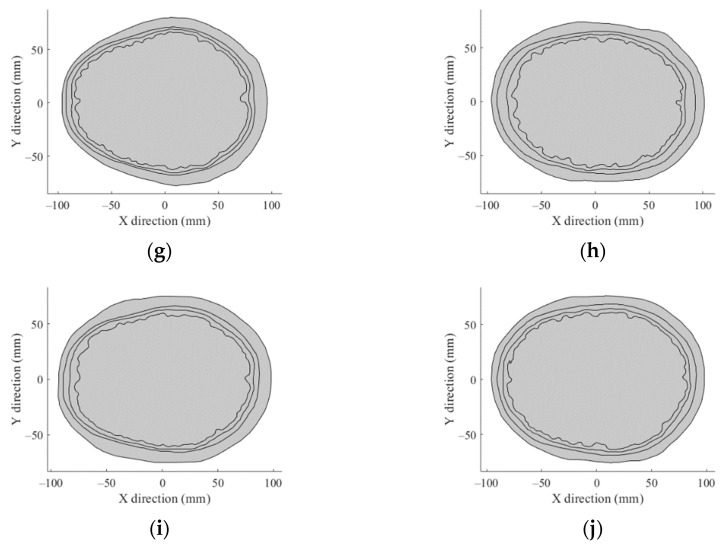
The 2D head models (**a**–**j**).

**Figure A2:**
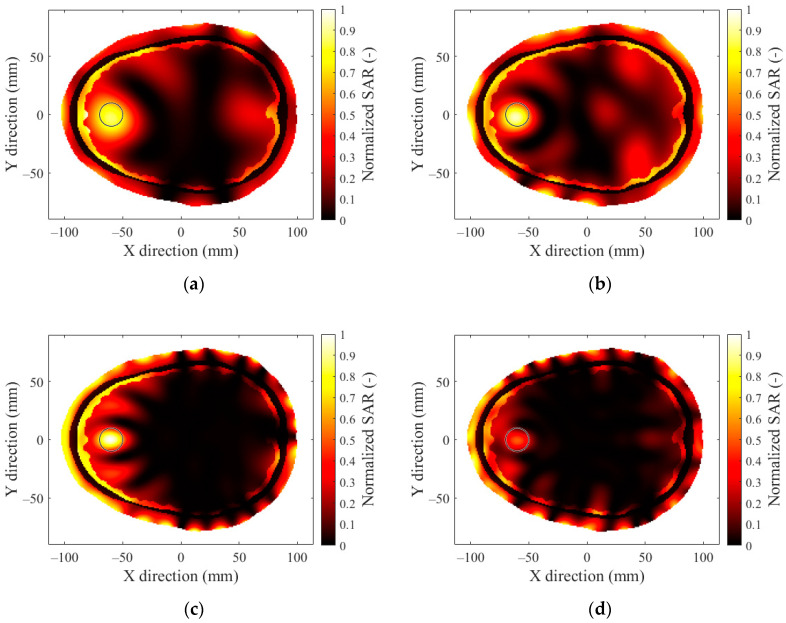
SAR distribution in the 2D head model obtained by a 24-antenna HT system at a frequency of (**a**) 434 MHz, (**b**) 650 MHz, (**c**) 915 MHz, and (**d**) 1150 MHz for a hyperthermia target diameter of 20 mm.

**Figure A3:**
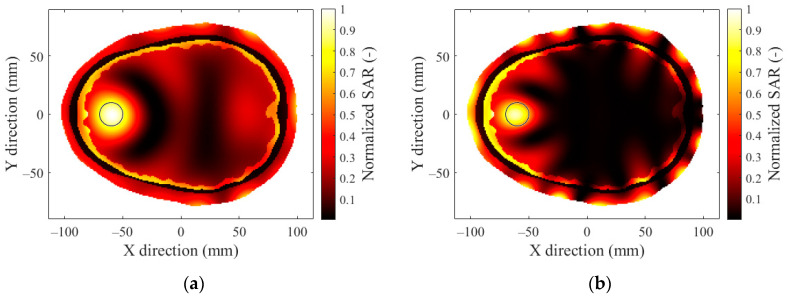

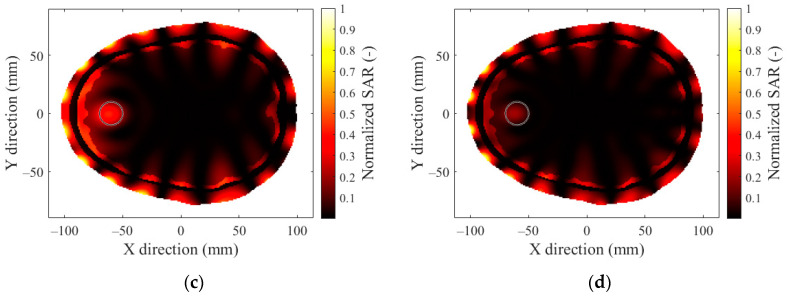
SAR distribution in the 2D head model obtained by a 16-antenna HT system at a frequency of (**a**) 434 MHz, (**b**) 650 MHz, (**c**) 915 MHz, and (**d**) 1150 MHz for a hyperthermia target diameter of 20 mm.

**Figure A4:**
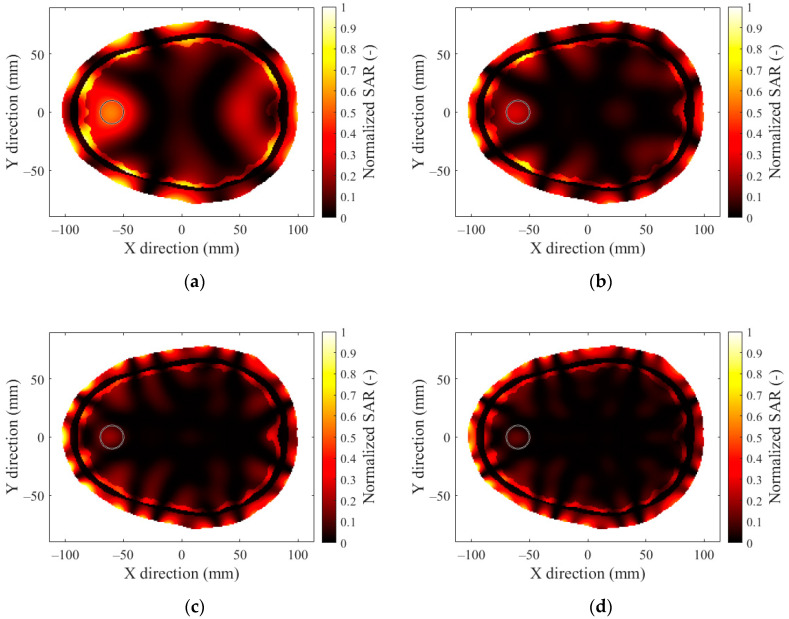
SAR distribution in the 2D head model obtained by an 8-antenna HT system at a frequency of (**a**) 434 MHz, (**b**) 650 MHz, (**c**) 915 MHz, and (**d**) 1150 MHz for a focus diameter of 20 mm.
